# Inhibition of eIF5A hypusination reprogrammes metabolism and glucose handling in mouse kidney

**DOI:** 10.1038/s41419-021-03577-z

**Published:** 2021-03-17

**Authors:** Marc Cougnon, Romain Carcy, Nicolas Melis, Isabelle Rubera, Christophe Duranton, Karine Dumas, Jean-François Tanti, Catherine Pons, Nicolas Soubeiran, Marina Shkreli, Thierry Hauet, Luc Pellerin, Sébastien Giraud, Nicolas Blondeau, Michel Tauc, Didier F. Pisani

**Affiliations:** 1 Université Côte d’Azur, CNRS, LP2M, Nice, France; 2 CHU Nice, Hôpital Pasteur 2, Service de Réanimation Polyvalente et Service de Réanimation des Urgences Vitales, Nice, France; 3 Université Côte d’Azur, INSERM, C3M, Nice, France; 4 Université Côte d’Azur, CNRS, INSERM, IRCAN, Nice, France; 5 Université de Poitiers, INSERM, IRTOMIT, CHU de Poitiers, La Milétrie, Poitiers, France; 6 CHU Poitiers, INSERM, IRTOMIT, Poitiers, France; 7 Université Côte d’Azur, CNRS, IPMC, Valbonne, France; 8 Present Address: Laboratory of Cellular and Molecular Biology, Center for Cancer Research, National Cancer Institute, Bethesda, MD 20892 USA

**Keywords:** Cell biology, Physiology

## Abstract

Inhibition of the eukaryotic initiation factor 5A activation by the spermidine analogue GC7 has been shown to protect proximal cells and whole kidneys against an acute episode of ischaemia. The highlighted mechanism involves a metabolic switch from oxidative phosphorylation toward glycolysis allowing cells to be transiently independent of oxygen supply. Here we show that GC7 decreases protein expression of the renal GLUT1 glucose transporter leading to a decrease in transcellular glucose flux. At the same time, GC7 modifies the native energy source of the proximal cells from glutamine toward glucose use. Thus, GC7 acutely and reversibly reprogrammes function and metabolism of kidney cells to make glucose its single substrate, and thus allowing cells to be oxygen independent through anaerobic glycolysis. The physiological consequences are an increase in the renal excretion of glucose and lactate reflecting a decrease in glucose reabsorption and an increased glycolysis. Such a reversible reprogramming of glucose handling and oxygen dependence of kidney cells by GC7 represents a pharmacological opportunity in ischaemic as well as hyperglycaemia-associated pathologies from renal origin.

## Introduction

The eukaryotic initiation factor eIF5A is the only protein known to be activated by the post-translational transformation of a specific lysine residue to hypusine through the so-called hypusination process^1^ which is mediated by the successive catalytic action of deoxyhypusine synthase (DHS) and deoxyhypusine hydroxylase^2^. A unique feature of eIF5A is that it seems necessary for translation of a limited number of mRNA through its ability to bind RNA in a sequence-specific manner^3^. eIF5A was also shown to be involved in the synthesis of only 5% of the total protein content in mammalian cells^4^ and particularly those containing polyproline sequences^5^. eIF5A is present as two isoforms: eIF5A1 which is ubiquitously expressed and eIF5A2 which is restricted to few tissues and is a hallmark of numerous types of cancer^6^. eIF5A and its hypusination step has been also described as involved in HIV-1 replication^7^, malaria disease^8^ or diabetes^9,10^. Interestingly, a strong relationship between hypoxia tolerance and the hypusination of eIF5A has been highlighted in drosophila model^11^. Considering this new concept we have recently shown in mammals that the specific inhibition of eIF5A hypusination by the spermidine analogue *N*1-guanyl-1,7-diamine-heptane (GC7)^12^ is able to enhance the ischaemic tolerance both at the cellular and tissue level in a rat kidney model of ischaemia/reperfusion injury^13^. Based on these results, we successfully used GC7 both in a preclinical model of kidney transplantation in pig^14^ and in a transient focal cerebral ischaemia (tFCI) model in mice^15^. Indeed, acute systemic administration of GC7 in the donor allowed better functional recovery of the kidney graft in the first case^13,14^ and reduced the infarct volume, and motor and cognitive post-stroke deficits in the second^15^. At the cellular level one of the first observations reported on cultured mouse kidney cells treated with GC7 was a metabolic shift from aerobic oxidative phosphorylation toward anaerobic glycolysis decreasing consequently oxygen consumption. In parallel, GC7-treated kidney cells displayed a mitochondrial remodeling characterized by a downregulation of respiratory chain complexes expression and activity while the ATP content remained high^13^. These observations have been recently corroborated in macrophages^16^.

In vertebrates the ischaemia tolerance depends in part upon their ability to urgently use anaerobic glycolysis to ensure their energy supply. This switch is time dependent and not adapted to an acute episode of ischaemia. Thus, the ischaemic tolerance reported through GC7 treatment suggests a pharmacological conditioning metabolic effect. Nonetheless, the molecular mechanism that support this process remains to be unravelled. Proximal cells manage glucose reabsorption while they do not use it as a source of energy for their own^17^. The transepithelial transport of glucose occurs in two steps^18^: (1) an apical membrane entry via SGLT1 and SGLT2, two sodium-linked co-transporters and (2) a basolateral export mediated by the facilitated glucose transporters GLUT1 and GLUT2. In this study, we aimed to understand how kidney cells conciliate glucose reabsorption function and anaerobic glycolysis under GC7 treatment. We finally propose that the pharmacological inhibition of eIF5A hypusination by GC7 reversibly reprogrammes the use of glucose by the proximal cells toward glycolysis parallelly to an impairment of renal glucose reabsorption due to GLUT1 misexpression. This leads to a deep and reversible cell metabolism remodeling allowing survival of kidney cells to oxygen deprivation. These results highlight a therapeutic opportunity in conditions of glucose homoeostasis unbalance.

## Materials and methods

### Reagents

*N*-guanyl-1,7-diaminoheptane (GC7) was synthesized by AtlanChim Pharma (Saint-Herblain, France) according to the methods described by Jasiulionis et al.^19^. Canagliflozin was purchased from Invitrogen (Cergy Pontoise, France). Culture media, buffer solutions, foetal bovine serum (FBS) and other culture reagents were from Sigma-Aldrich (Saint-Quentin Fallavier, France).

### Animals

The experiments were conducted in accordance with the French and European regulations (2010/63/EU directive) for the care and use of research animals and were approved by national experimentation committees (MESR No.: APAFIS#22670-2019101811258232).

Ten-week-old C57BL/6 male mice from Janvier Laboratory (France) were maintained at housing temperature (22 °C) and 12:12-h light–dark cycles, with ad libitum access to food and water. Mice were daily treated with GC7 (3 mg/kg in saline solution, intraperitoneal injection, *n* = 12, “GC7” group) or with vehicle only (saline solution NaCl 0.9% w/v, intraperitoneal injection, *n* = 12, “ctrl” group) for 3 days. Animals analysis was not blinded.

### Cell culture

Renal proximal convoluted tubule cells (PCT) were obtained from primary cultures of murine proximal tubule segments, immortalized with pSV3neo vector and were cultured as previously described^20,21^. Cultures were classically maintained in a 5% CO_2_/95% air water-saturated atmosphere in M1 medium (DMEM/F12, Glutamine, SVF, EGF, T3, dexamethasone, ITS, G418). All experiments were performed the day after cell confluence.

### Cell metabolism analysis

The oxygen consumption rate (OCR) and extracellular acidification rate (ECAR) of PCT cells were determined using an XF24 Extracellular Flux Analysedr (Seahorse Bioscience). Uncoupled and maximal OCR were determined using oligomycin (1.2 µM) and FCCP (1 µM). Rotenone and Antimycin-A (2 µM each) were used to inhibit mitochondrial respiration. All parameters were calculated as described previously^22^.

### Cell survival analysis

The Fluorescent LIVE/DEAD® Cell Viability/Cytotoxicity Assay Kit (Invitrogen, France) was used on PCT cells (24-well plates), according to the manufacturer’s protocol. Fluorescent micrographs were recorded using an observer D1 microscope (Zeiss, Germany) and analysed using imageJ software.

### Protein analysis

Whole proteins from cells and tissues were prepared using TNET lysis buffer (25 mM Tris-Cl (pH 7.4), 100 mM NaCl, 1 mM EDTA, 1% Triton X-100, 0.5% Nonidet P40, 1× protease inhibitor cocktail and 1× Phosphostop mix (Roche Diagnostics, Meylan, France)). In addition, tissues were solubilized using a Precellys tissue homogenizer in ice‐cold buffer and using CK14 beads (Bertin Technologies, Ozyme). For membrane protein-enriched lysates, cells were washed twice with cold phosphate-buffered saline (PBS), once with cold water and finally incubated in hypotonic buffer (Tris 10 mM, EDTA 1 mM, pH 7.5, 1× protease inhibitor cocktail) 10 min on ice. Cells were scratched and disrupted using a 25G needle. Crude lysate was centrifuged first at 2000*g* (10 min, 4 °C) to eliminate undisrupted cells and nuclei, and then at 10,000*g* (30 min, 4 °C). Pellet containing membranes was re-suspended in hypotonic buffer containing protease inhibitors.

Protein concentration was evaluated by BCA assay (PIERCE, Thermo Scientific, France) and blotted using the SDS-PAGE basic protocol. Primary antibody incubation was performed overnight at 4 °C (eIF5A, Abcam #32443; GLUT1, Abcam #ab54460; GLUT2, Cell Signaling #54460; hypusine-eIF5A^13^; β-actin, Sigma # A5441) and then with adequate HRP-conjugated secondary antibodies (Jackson ImmunoResearch, Interchim, France) (30 min, 1:10,000, RT). Detection was performed using Immobilon Western Chemiluminescent HRP Substrate (Millipore, Molsheim, France) and Fuji apparatus. Band intensities were evaluated using PCBas Software.

Lactate dehydrogenase (#K2228, APExBIO, Clinisciences) and glyceraldehyde 3-phosphate dehydrogenase (#K680, BioVision, Clinisciences) cells content were evaluated using activity assay and following the manufacturer’s instructions.

### Biochemical parameter analysis

Urinary creatinine was assayed by colorimetric reaction (NaOH 0.75N/Picric acid 0.04 M V/V) detected at 520 nm. Glucose was evaluated directly in plasma, urine and cell supernatant using Glucose-Glo Assay (Promega) and following the manufacturer’s instructions. Cell glucose efflux was directly measured in glucose-free media at indicated time. Cell glucose consumption was evaluated by the assay of glucose decrease in full media containing 4.5 g/L glucose at indicated time and normalized by glucose efflux measured at the same time.

Glucose uptake was measured using 2-deoxy-d-[^3^H]-glucose (2-DG). After two washes with PBS, cells were incubated 10 min with 100 µM 2-DG and 1 µCi 2-deoxy-d-[^3^H] glucose. Culture plates were put on ice and rinsed with ice-cold PBS. Cells were scraped in 0.5 N NaOH, neutralized with 0.5 N HCl and 2-DG uptake was measured by liquid scintillation counting of cell lysate with a beta-counter.

Ion concentrations (Cl^−^, lactate, Na^+^, K^+^) were evaluated by ion chromatography analysis. All biological samples (plasma, urine and cell supernatant) as well as ion standard solutions were previously deproteinized by addition of acetonitrile (dilution 1:1 volume). Samples were strongly mixed and centrifuged at 12,000*g* (10 min at 4 °C). Ion concentrations of the supernatant were determined using an ion chromatography Dionex ICS-5000 plus system (Thermo Scientific). The system included an autosampler, pumps, eluent generator and conductivity detectors. The system was equipped with two eluent generator cartridges (Dionex EGC500KOH; Dionex EGC500MSA), an anion column (IonPac CS17, 2 mm) and a cation column (IonPac AS-11 HC, 2 mm). Ion concentrations were determined using Chromeleon software (Thermo Scientific) by measuring surface area of the peaks and were compared to the corresponding ion standard profiles.

### mRNA analysis

Procedures follow MIQE recommendations^23^. Total RNA was extracted using TRIzol (Invitrogen) according to the manufacturer’s instructions. In addition, tissues were solubilized using a Precellys tissue homogenizer in TRIzol reagent and using CK14 beads. Reverse transcription-polymerase chain reaction (RT-PCR) was performed using M-MLV-RT (Promega). SYBR qPCR premix Ex TaqII from Takara (Ozyme, France) was used for quantitative PCR (qPCR), and assays were run on a StepOne Plus ABI real-time PCR machine (PerkinElmer Life and Analytical Sciences, Boston). The expression of selected genes was normalized to that of the TATA-box-binding protein (TBP) and β-actin housekeeping genes, and then quantified using the comparative-ΔCt method. Primer sequences are available upon request.

### Histology and immunohistochemistry

Freshly sampled tissues were fixed in 10% buffered formalin overnight at RT and then paraffin embedded. Embedded tissues were cut in 5 μm sections and dried 30 min at 55 °C. For immunohistochemistry, sections were then deparaffinized in xylene, rehydrated through alcohol, and washed in PBS. Antigen retrieval was performed using Vector unmasking reagent (Vector Laboratories). Sections were blocked with MOM blocking solution (MOM kit, Vector Laboratories), and then incubated with mouse monoclonal primary antibody anti-GLUT1 (Abcam, #ab40084) diluted in MOM diluent for overnight at 4 °C. Detection was performed with Alexa647-conjugated donkey anti-mouse secondary antibody (Thermo Fisher Scientific). GLUT1-stained kidney sections were sequentially scanned for far red signal allowing imaging of a large area of the sections. GLUT1 signal analysis was then performed using ImageJ software (Supplementary Fig. 5).

### Statistical analysis

Data were analysed using GraphPad Prism 6 software. Calorimetry data were analysed using two‐way ANOVA multiple comparison and activity using multiple *t*-test. Other data were analysed by Mann–Whitney (two groups) or Kruskal–Wallis (more than two groups) test to assess statistical differences between experimental groups. Cell sample size was chosen based on the need for statistical power. Animal cohort sizes have been determined using G*Power and groups were randomized^24^. Differences were considered statistically significant with *p* < 0.05. Data were displayed as the scatter plot of independent values and group mean values ± SD.

## Results

### GC7 promotes anaerobic glycolysis

**Fig. 1:**
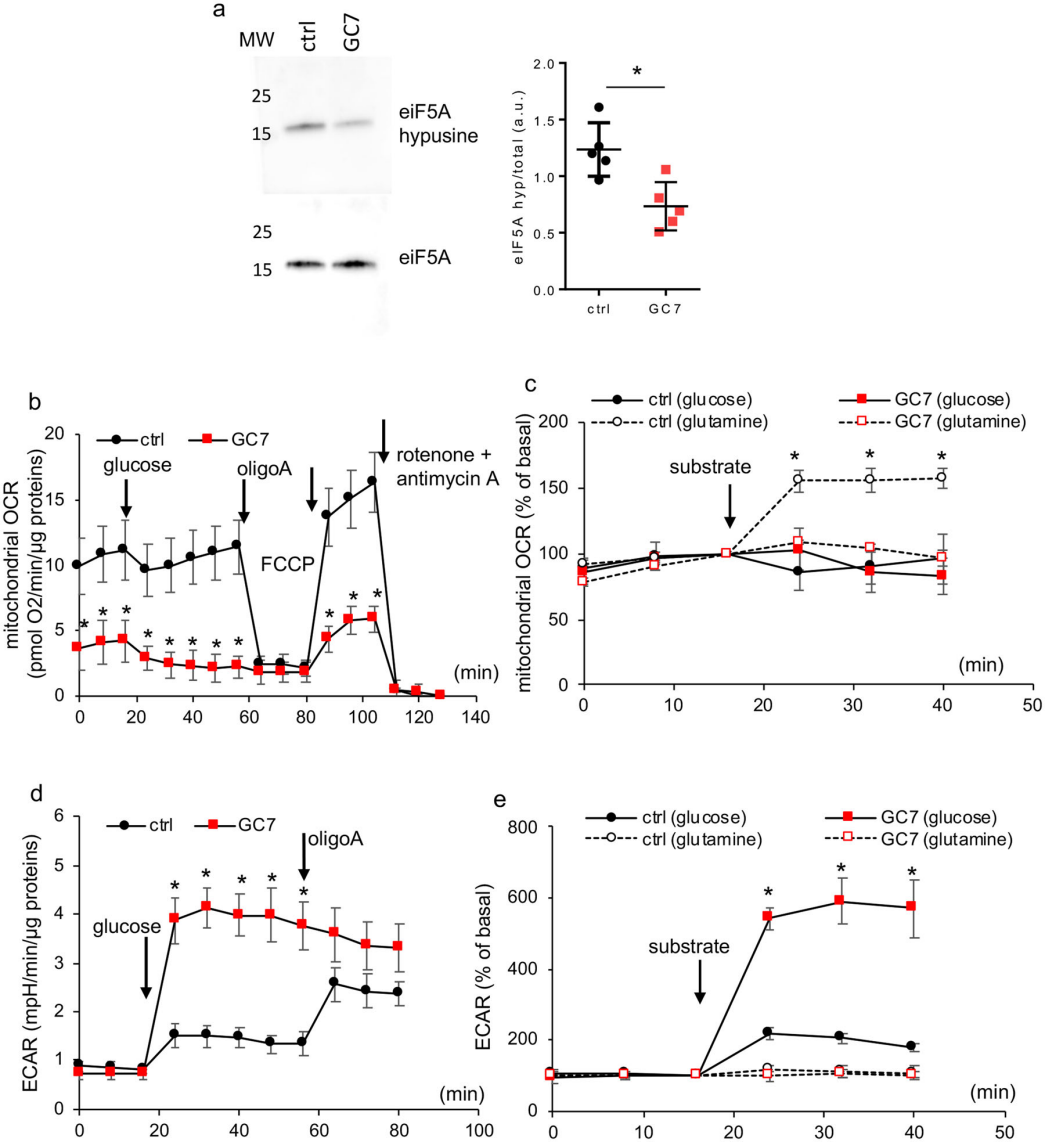
GC7 induces a switch in cell metabolism and substrate preference. Confluent PCT cells were treated or not with 30 µM GC7 for 24 h and then used for different analysis. **a** Representative western blot displaying inhibition of eiF5A hypusination and ratio between optical density of hypusine and eIF5A band. MW molecular weight. **b**–**e** Cells were analysed using Seahorse technology to evaluate oxygen consumption (**b**, **c**) and extracellular acidification (**d**, **e**) after addition of 10 mM glucose (**b**–**e**) or 2 mM glutamine (**c**, **e**). Addition of other compounds are indicated. Dots displayed mean ± SD of three independent experiments. *p < 0.05 evaluated using Mann–Whitney (**b**, **d**) or Kruskal–Wallis (**c**, **e**) tests.

We firstly show that GC7, which induced a decrease of eIF5A hypusination in the kidney in vivo^13^, had the same effect in our in vitro model of PCT cells. Figure 1a shows that GC7 treatment (24 h, 30 µM) induced a decrease of eIF5A hypusination in these cells issued from proximal tubules^25^. The dose of 30 µM we used was previously shown to be the lowest dose with a maximal protective effect on the anoxia induced cell death^13^. Since the metabolic switch observed involves a mitochondrial downregulation^13^ we investigated how GC7-induced switching from oxidative phosphorylation to glycolysis affects glucose and the overall metabolism of PCT cells treated 24 h with GC7. Mitochondrial OCR of GC7-treated cells maintained in glucose free medium was very low and not modulated by addition of 10 mM glucose. By contrast, PCT control cells exhibited a higher rate of basal O_2_ consumption but remained also insensitive to the addition of glucose (Fig. 1b). Using a substrate free medium, we demonstrated that PCT control cells increased their mitochondrial O_2_ consumption upon addition of 2 mM glutamine instead of glucose, and that GC7-treated cells were unable to increase their O_2_ consumption whatever the substrate used (Fig. 1c). The pH analysis of both types of cells revealed an increase in extracellular acidification upon glucose addition (Fig. 1d, e) and not with that of glutamine (Fig. 1e). However, this increase was threefold higher in GC7-treated cells (Fig. 1d, e). This metabolic analysis demonstrated that GC7 treatment switched PCT cells from amino acid-dependent oxidative phosphorylation and used glucose instead of glutamine as an energetic substrate through the anaerobic glycolysis pathway.

### GC7 enhances glucose consumption

**Fig. 2:**
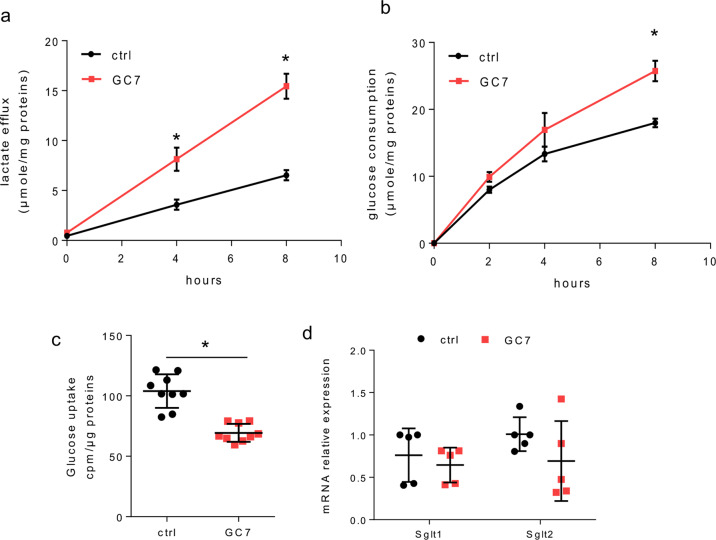
GC7 increases glucose consumption and lactate efflux. Confluent PCT cells were treated or not with 30 µM GC7 for 24 h. **a** Measurement of lactate media content related to whole-cell proteins content. **b** Evaluation of cell glucose consumption corresponding to the difference between measurement of the media glucose content decrease (in the presence of glucose in media) and media glucose content increase (after glucose deprivation). Dots displayed mean ± SD of three independent experiments. *p < 0.05 evaluated using Mann–Whitney (**a**, **b**) tests. **c** Glucose uptake measured using 2-deoxy-d-[^3^H]-glucose. Results displayed cpm of 2-deoxy-d-[^3^H]-glucose measured in cell lysate. **d** RT-qPCR analysis of glucose transporters from the SGLT family. Graphic displayed the scatter plot of 9 (**c**) or 5 (**d**) independent values and mean ± SD. *p < 0.05 evaluated using Mann–Whitney test.

We then analysed the kinetic of lactate efflux and glucose consumption in PCT cells after 24 h of GC7 treatment (Fig. 2a, b). Correlatively to media acidification analysis, the lactate efflux was increased (threefold more at 8 h) in GC7-treated cells (Fig. 2a). This was associated to a significant increase in glucose consumed by the cells (Fig. 2b). Looking for a modification in glucose flux, we analysed the glucose uptake using 2-deoxy-d-[^3^H] glucose. Such analysis revealed a significant decrease of glucose uptake in GC7-treated cells (Fig. 2c). This modification was not linked to a decrease in Na^+^-dependent glucose transporters SGLT1 and SGLT2 (responsible of glucose uptake from nephron lumen by proximal cells in vivo) mRNA expression (Fig. 2d).

### SGLT2 glucose entry is essential to GC7-treated cell metabolism and survival

**Fig. 3:**
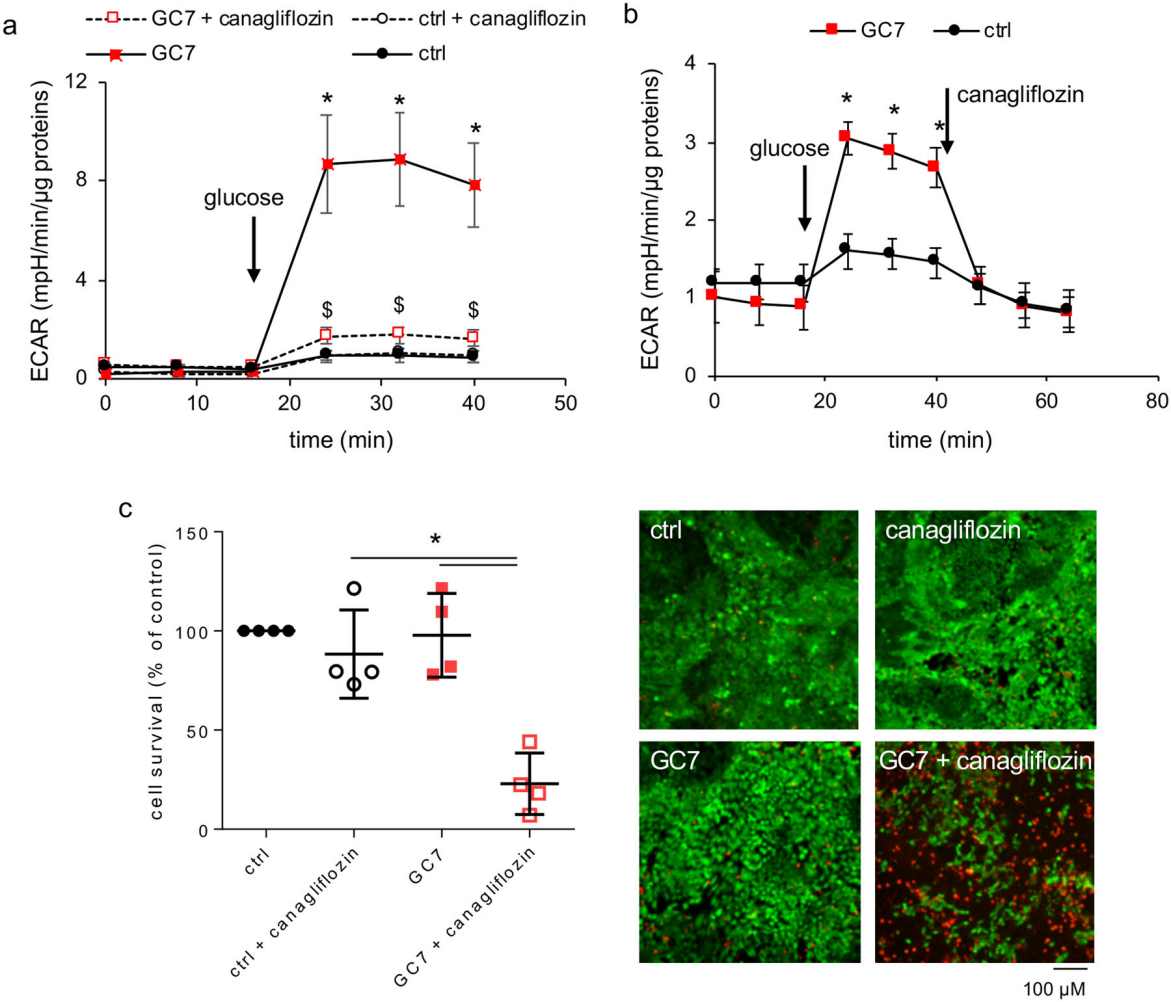
SGLT2 glucose entry is essential to GC7-treated cell metabolism and survival. **a**, **b** Confluent PCT cells were treated or not with 30 µM GC7 for 24 h and then glycolysis in response to glucose addition (10 mM) was evaluated using Seahorse technology. Canagliflozin (10 µM) was added 4 h before analysis or along analysis as indicated. Dots displayed mean ± SD of three independent experiments. **c** Mortality evaluation using live/dead cells fluorescence assay of PCT cells co-treated with 10 µM canagliflozin and 30 µM GC7. Representative pictures for each group are displayed and show live (green, calcein-FITC) and dead (red, ethidium bromide homodimer) cells. Scale bar is indicated. Graphic displayed scatter plot of four independent values and mean ± SD. *p < 0.05 evaluated using Kruskal–Wallis (**a**, **c**) or Mann–Whitney (**b**) tests.

As glucose enters proximal cells mainly by the Na^+^-glucose transporter SGLT2, and in a lower extent by SGLT1 (ref. ^18^) in proximal tubule cells, we used the SGLT2 inhibitor canagliflozin to disrupt this crucial step in control and GC7-treated cells (30 µM, 24 h). Pretreatment (4 h) as well as acute treatment with 10 µM canagliflozin blunted medium acidification of PCT cells upon addition of glucose and consequently anaerobic glycolysis (Fig. 3a, b). Moreover live/dead analysis after 24 h of treatment with 10 µM canagliflozin revealed a cytotoxic effect of the SGLT2 inhibition leading to a mortality of approximately 75% in GC7-treated cells, but not in control canaglifozin-treated PCT cells and untreated cells that both remained alive (Fig. 3c). Thus, glucose entry via SGLT2 transporter is required for survival of GC7-treated PCT cells contrary to control cells.

### Glycolysis is the exclusive energy source in GC7-treated cells

**Fig. 4:**
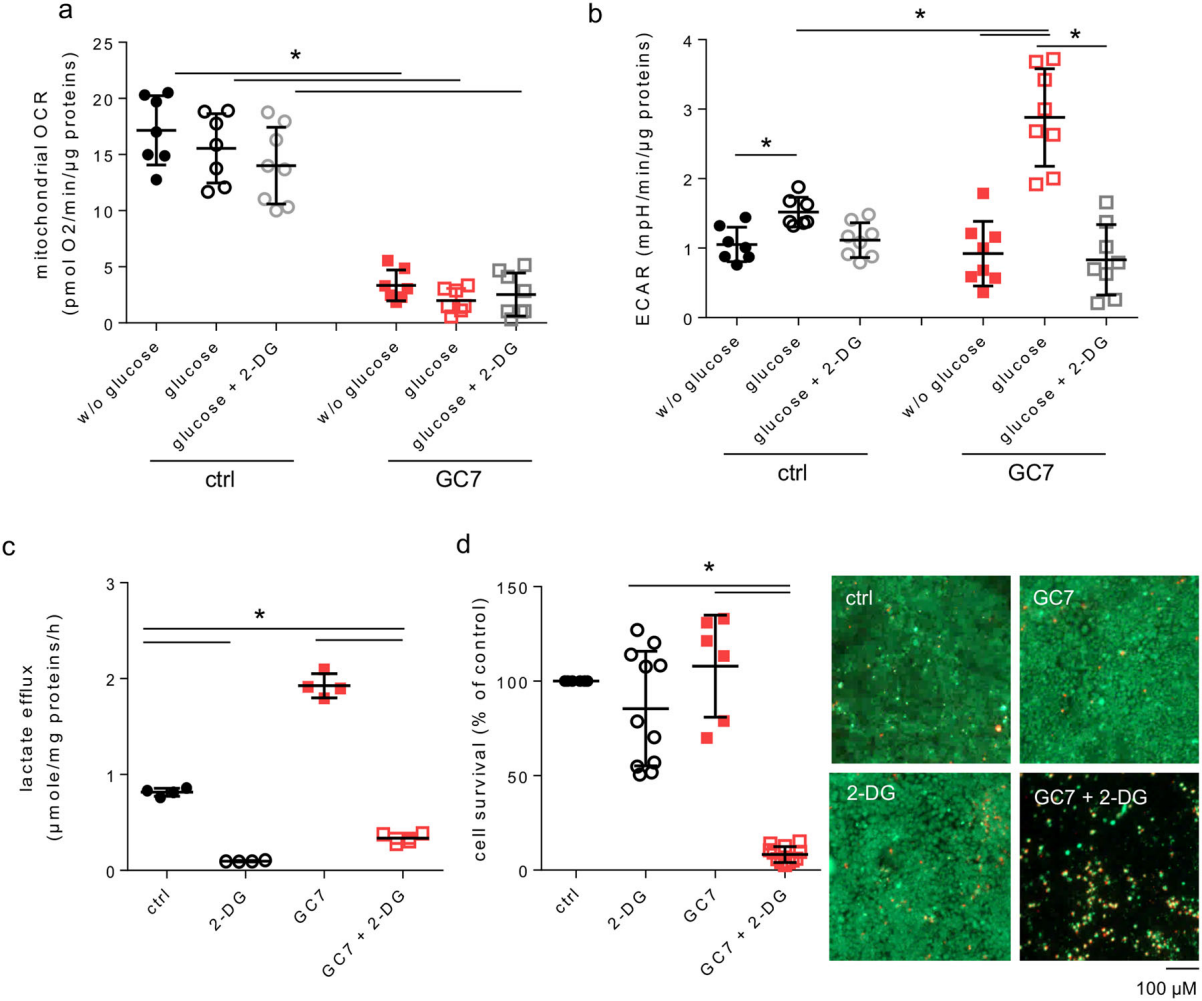
Glycolysis is essential to GC7-treated cell metabolism and survival. **a**, **b** Confluent PCT cells were treated or not with 30 µM GC7 for 24 h and then (**a**) oxygen consumption (OCR) and (**b**) extracellular acidification (ECAR) rates were measured using Seahorse technology. Analysed was performed first without glucose and then glucose (10 mM) and 2-deoxy-glucose (2-DG, 25 mM) were sequentially added as indicated. **c** Confluent PCT cells were treated or not with 30 µM GC7 for 24 h and by 25 mM 2-DG the last 8 h. Graphic displayed measurement of lactate media content after 8 h related to whole-cell proteins content. **d** Confluent PCT cells were treated or not with 30 µM GC7 and 25 mM 2-DG for 24 h. Mortality was evaluated using live/dead cells fluorescence assay. Representative pictures for each group are displayed and show live (green, calcein-FITC) and dead (red, ethidium bromide homodimer) cells. Scale bar is indicated. Graphics displayed scatter plot of 8 (**a**, **b**), 4 (**c**) and 11 (**d**) independent values and mean ± SD. *p < 0.05 evaluated using Kruskal–Wallis.

To evaluate the importance of glucose as energy source for GC7-treated PCT cells, we treated or not PCT cells with GC7 (30 µM, 24 h) and analysed their metabolism by sequential addition of glucose and 2-deoxy-glucose (2-DG) (Fig. 4a, b). As previously shown, the respiratory capacity of PCT cells was not modified after glucose addition and was insensitive to 2-DG (Fig. 4a). Differently, glucose addition increased slightly ECAR in control cells and strongly in GC7-treated ones whereas 2-DG inhibited it close to the basal level (Fig. 4b). This is confirmed by the measurement of lactate efflux that showed that 2-DG treatment inhibited the release of lactate in control and GC7-treated PCT cells (Fig. 4c). Thus, the weak glycolytic activity displayed by control cells was notably increased by GC7. Survival of PCT cells, which displayed a basal glycolysis, was not affected by addition of 2-DG (Fig. 4d). Indeed, these cells use glutamine-fuelled oxidative phosphorylation as energy source (Fig. 1). By contrast, GC7-treated cells displayed more than 90% of mortality under 2-DG treatment (Fig. 4d), demonstrating clearly that glucose-dependent glycolysis is the unique energy source of the GC7-treated cells.

### GC7 inhibits glucose efflux in proximal cells and represses GLUT1 expression

**Fig. 5:**
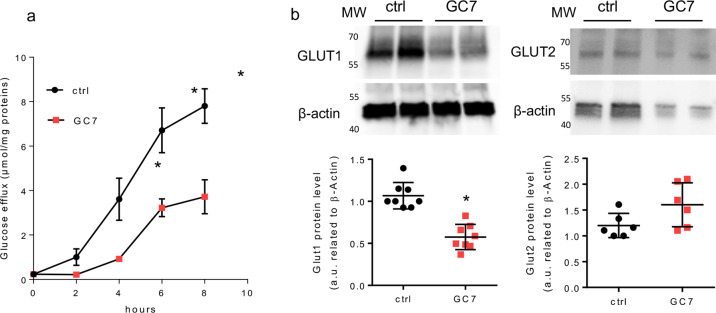
GC7 modulates glucose flux in PCT cells. Confluent PCT cells were treated or not with 30 µM GC7 for 24 h and then used for different analysis. **a** At the end of treatment cells were deprived in glucose and the efflux of glucose was measured in the next 8 h. Dots displayed mean ± SD of three independent experiments. **b** Western blot analysis of GLUT1 and GLUT2 expressions in membrane enriched protein lysate from PCT cells. β-Actin was used as loading control. MW molecular weight. *p < 0.05 evaluated using Mann–Whitney test.

As proximal cells mediate glucose reabsorption into the interstitial milieu in vivo, we looked for their in vitro glucose efflux capacity. As shown in Fig. 5a, the glucose efflux was decreased in GC7-treated PCT cells and reaches a more than twofold difference after 8 h of treatment. In proximal cells the glucose efflux corresponds to a passive transport through both the GLUT1 and GLUT2 facilitated transporters expressed at the basolateral membrane, so we analysed the effect of GC7 on their expression. The GLUT1 protein expression was impaired in GC7-treated PCT cells whereas that of GLUT2 was not significantly modified (Fig. 5b).

### LDH and PDH activities are not involved in the GC7-induced metabolic shift

The lack of oxidative phosphorylation could be due to a high activity of lactate dehydrogenase (LDH)-reducing pyruvate availability to enter mitochondria. We did not find any difference between untreated and GC7-treated cells in LDH activity in whole-cell lysates (Supplementary Fig. 1a). Moreover, we used oxamate, a noncompetitive inhibitor of LDH. Oxamate treatment (100 mM) of control and GC7-treated cells inhibited strongly the lactate efflux (Supplementary Fig. 1b) and in a lesser extent media acidification (Supplementary Fig. 1c). Analysis of oxygen consumption did not reveal any effect of oxamate in control and GC7-treated cells (Supplementary Fig. 1d). Thus, increasing pyruvate availability in PCT cells did not favour its use by mitochondria. Moreover, oxamate treatment did not lead to cell death neither in control nor in GC7-treated cells (Supplementary Fig. 1e), suggesting that cell survival was not affected under oxamate treatment contrarily to canaglifozin or 2DG. These results demonstrate that GC7-induced metabolic shift was not linked to LDH activity.

Pyruvate dehydrogenase (PDH), a key enzyme of OXPHOS, can be inactivated by multiple phosphorylations of serine residues (Ser232, Ser239 and Ser300) catalysed by pyruvate dehydrogenase kinases (PDK). In control PCT cells PDH was highly phosphorylated and these phosphorylations were decreased in the presence of 5 mM dichloroacetate (DCA) (Supplementary Fig. 2a) an inhibitor of PDK, which maintained PDH in an active form. Surprisingly, in GC7-treated cells all residues were found dephosphorylated close to the values found after DCA treatment (Supplementary Fig. 2a). As DCA promotes pyruvate entry into the Krebs cycle, we checked for oxygen consumption in GC7-treated and untreated cells and submitted to DCA (5 mM, 24 h). As expected, DCA increased OCR in control cells allowing activation of PDH. In contrast, no change was observed in GC7-treated cells (Supplementary Fig. 2b), demonstrating that pyruvate cannot be used as substrate for oxidative phosphorylation and is preferentially reduced in lactate. In the same way no change by DCA treatment was found in lactate efflux (Supplementary Fig. 2c) as well as in the cell survival rate (Supplementary Fig. 2d) of GC7-treated cells.

### The GC7-induced metabolic switch is fully reversible

**Fig. 6:**
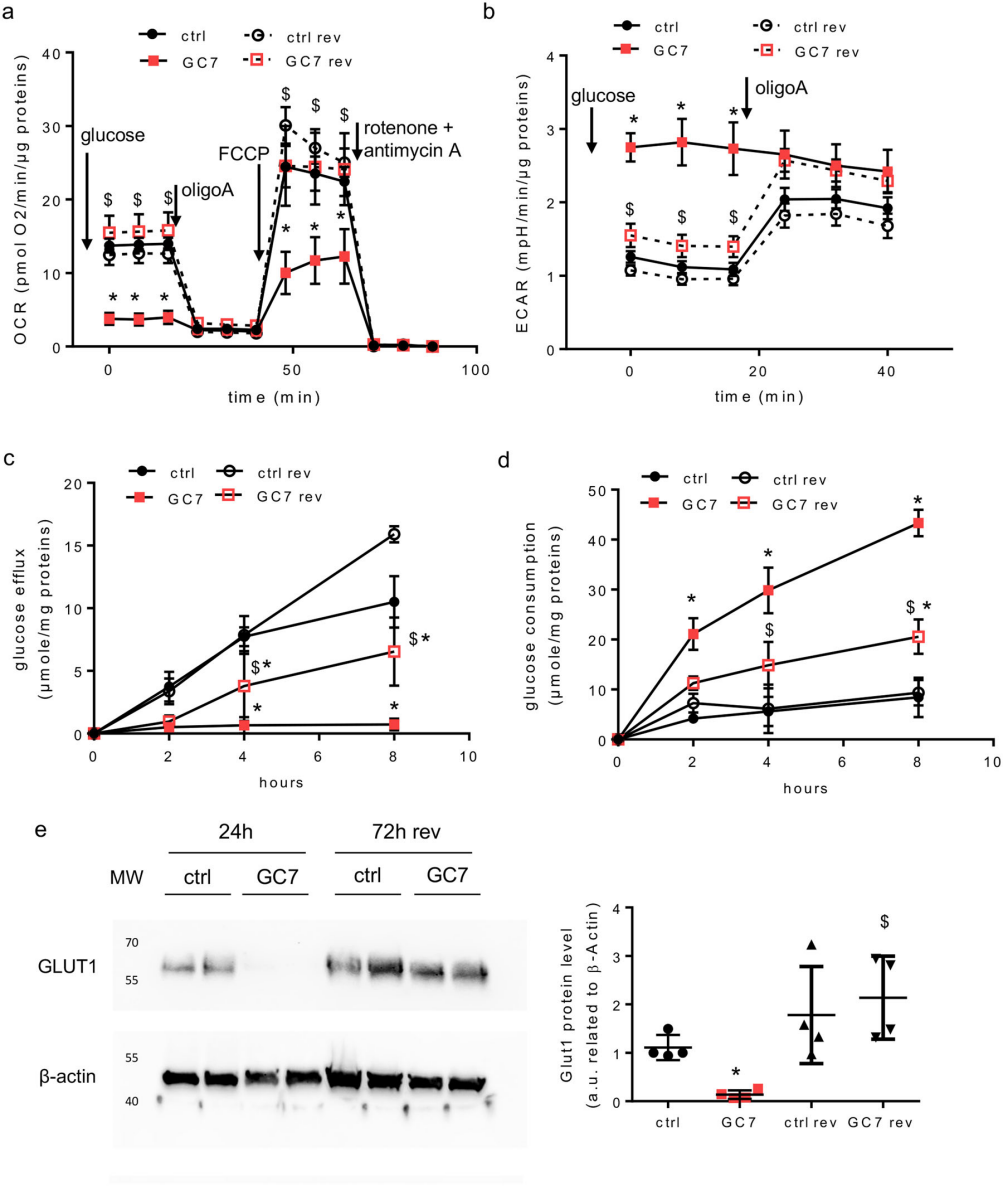
The effect of GC7 is fully reversible. Confluent PCT cells were treated or not with 30 µM GC7 for 24 h and then GC7 was removed and cells were maintained for an additional period of 72 h and analysed. **a**, **b** Cells were analysed using Seahorse technology to evaluate oxygen consumption (**a**) and extracellular acidification (**b**) in the presence of 10 mM glucose. Addition of other compounds are indicated. Dots displayed mean ± SD of three independent experiments. *p < 0.05 evaluated using Mann–Whitney. **c** At the end of treatment cells were deprived in glucose and the efflux of glucose was measured in the next 8 h. **d** Measurement of cell glucose consumption corresponding to the difference between measurement of the media glucose content decrease (in the presence of glucose in media) and media glucose content increase (after glucose deprivation). Dots displayed mean ± SD of three independent experiments. *p < 0.05 evaluated using Mann–Whitney tests. “Ctrl rev” are control cells at 72 h without GC7 treatment and “GC7 rev” correspond to cells 72 h after GC7 removal. “Ctrl” and “GC7” are cells analysed after 24 h. **e** Western blot analysis of GLUT1 in membrane-enriched protein lysate from PCT cells following a 24 h GC7 treatment (30 µM) and after 72 h of GC7 washout (72 h rev). β-actin was used as a loading control. MW molecular weight. *p < 0.05 evaluated using Mann–Whitney test.

We next analysed the recovery of the OXPHOS pathway following GC7 removal. Confluent PCT cells were treated for 24 h with 30 µM GC7. Afterward GC7 was removed and cells were maintained in culture medium for an additional period of 72 h. Seahorse experiments revealed that GC7 pre-treated PCT cells recovered a basal OCR close to the one measured in control condition (Fig. 6a) in the presence of 10 mM glucose. In the same way the ECAR of these cells returned to the values observed in control cells (Fig. 6b). This indicates that PCT cells are able to recover an OXPHOS, confirming the reversion of the GC7 effect. From a functional point of view, Fig. 6c shows that 72 h after GC7 removal the glucose efflux of PCT cells, which is fully impaired by GC7, is hugely recovered. Parallelly the glucose consumption tends to return toward the value observed in control cells (Fig. 6d). These data demonstrate that the mitochondrial “silencing” induced by GC7 is reversible as far as both the metabolic and functional features of PCT cells are concerned. This conclusion was reinforced by the fact that GLUT1 expression which is fully repressed by GC7 is back following the recovery period (Fig. 6e).

### Impact of GC7 treatment on mice physiological parameters

**Fig. 7:**
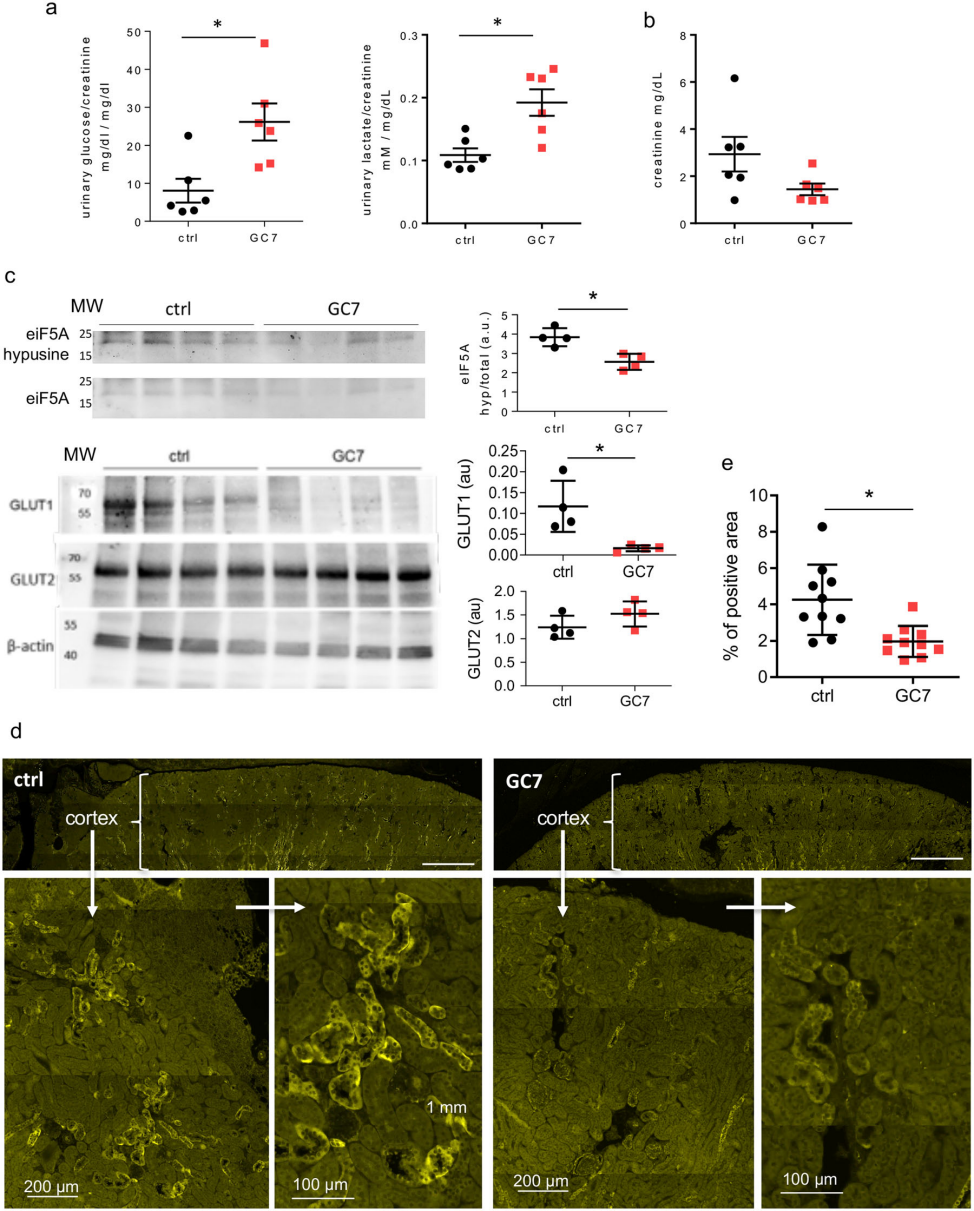
Impact of a 3-days GC7 treatment on kidney glucose metabolism. Mice were treated 3 days with GC7 (3 mg/kg, i.p., “GC7” group) or vehicle only (NaCl, “ctrl” group) and metabolic parameters were analysed at the end of the treatment. **a** Glucosuria (glucose/creatinine) and lactaturia (lactate/creatinine) measured in bladder urine. **b** Urinary creatinine (mg/dL). **c** Hypusination of eIF5A and GLUT1 and GLUT2 protein levels evaluated by western blot using four kidney protein extract for each group. MW molecular weight. Graphic displayed hypusine/total eIF5A ratio and GLUT/β-actin ratio using optical density band values. **d** Mosaic reconstruction of whole-kidney sections from untreated and GC7-treated mice displaying GLUT1 signals in the cortical region. Magnification of a representative cortical regions from each group. Scale bars are indicated. **e** Quantification of GLUT1 immunofluorescence. Graphics displayed the scatter plot of 4 (**c**), 6 (**a**, **b**) or 10 (**e**) independent values and mean ± SD. *p < 0.05 evaluated using Mann–Whitney test.

We looked in vivo for the metabolic and main physiological effects associated to the overall modifications observed in vitro. Mice were treated daily with GC7 (IP, 3 mg/kg/day; “GC7” group) or with vehicle only (NaCl 0.9% w/v; “ctrl” group) for 72 h, according to the protocol demonstrated to have beneficial effect in rodent and pig models^13–15^. No difference was found between control and GC7 treated mice for sodium, potassium and chloride ion contents in plasma and urine (Supplementary Fig. 3a). Interestingly, while glucose and lactate plasma levels were unchanged upon GC7 treatment (Supplementary Fig. 3b), urinary glucose and lactate were significantly higher in GC7 compared to control mice when reported to urinary creatinine (Fig. 7a). Moreover, urinary creatinine was not significantly different between the two groups suggesting an unaltered renal function (Fig. 7b). To verify that these phenotypes were linked to GC7 effect, we confirmed that eiF5A hypusination was decreased in the kidney after 3 days of GC7 treatment (Fig. 7c).

### GLUT1 expression is impaired in kidneys from GC7-treated mice

As the increase in glucosuria and lactaturia found in mice could be due to the altered expression of glucose transporters shown in vitro (Fig. 6e), we studied the effect of GC7 treatment on the expression of glucose transporters in mice kidneys. As observed in PCT cells we retrieved a decrease in the protein level of GLUT1, but not of GLUT2, in whole-kidney protein extract from GC7-treated mice (Fig. 7c). To confirm the GLUT1 misexpression in kidney, we evaluated its expression in the renal cortical region by immunohistochemistry using sagittal kidney sections from control and GC7-treated mice. As displayed in Fig. 7d, the expression of GLUT1 is clearly noticeable in control kidneys at the basolateral membrane of the tubules while its expression is impaired in term of labelling intensity (Fig. 7d) and number of positive tubules in kidneys from GC7-treated mice as evaluated by quantifying the labelled areas of each sections (Fig. 7e and Supplementary Figs. 4 and 5).

## Discussion

**Fig. 8:**
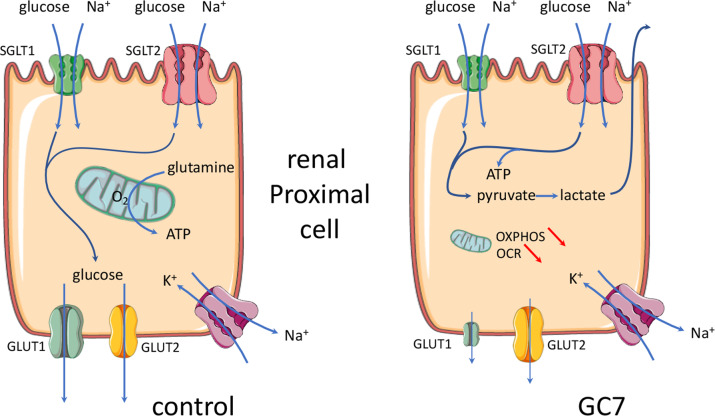
Schema of GC7 effects on glucose handling in proximal cells. Schematic representation of normal and GC7-treated proximal cells regarding the glucose handling. GC7 represses the oxidative phosphorylation mediated by the mitochondrial function and enforces the cell to shift toward a glycolytic pathway to maintain energetic status. The apical flux of glucose mediated by the SGLT2 transporter is thus necessary to feed this pathway and cell survival becomes then largely independent of ambient oxygen. This reversible shift is accompanied by a strong inhibition of the glucose reabsorption through the basolateral membrane due to the downregulation of the facilitated transporter GLUT1.

We have previously demonstrated that inhibition of eIF5A hypusination, through conditioning treatment with the spermidine analogue GC7, led to anoxic tolerance in kidney cells^13,14^ and neurons^15^ both in vitro and in vivo. Tolerance to anoxia due to GC7 treatment has been shown to be linked to an inhibition of mitochondrial activity together with an increase in anaerobic glycolysis that allowed cell survival by maintaining basal ATP synthesis and membrane integrity. Here we are advancing in understanding this mechanism by demonstrating a phenotypical switch of the proximal tubule cells treated with GC7, from a full functional state to a survival state based on the fate of glucose. In in vivo control condition proximal convoluted tubular cells uptake glucose via SGLT2 transporters family in the S1 segment and drive it out to the interstitial space in vivo using the high-affinity GLUT1 and low-affinity GLUT2 facilitated glucose transporters^26^. Moreover, these cells do not use this glucose as energy source for their own and rather generate ATP from oxidative phosphorylation of glutamine^17,27^. Herein, we demonstrate that GC7 treatment inhibits the efflux of glucose (i.e. glucose reabsorption) and mitochondrial oxidative phosphorylation, which leads to the establishment of anaerobic glycolysis to ensure the energy demand necessary for cell survival (Fig. 8). Interestingly, while under GC7 treatment the cells were unable to use another source of energy than glucose, this capacity was not lost as the cells retrieve normal metabolism and function after GC7 removal^13,14^.

This functional and metabolic shift allows cells to become transiently independent of the environmental oxygen and thus to resist to the deep decrease in oxygen concentration resulting either from anoxia in vitro or ischaemia in vivo^13^. With regard to kidney transplant where the GC7-conditioning lasts a couple of days after treatment, cells resist to the high deleterious reoxygenation step characterizing the reperfusion step in vivo^14^. Anoxia tolerance of proximal tubular cells is a crucial challenge as they are highly dependent on oxidative metabolism and they do not use glucose as energy source^17^. Thus, opportunistically the GC7 takes advantage of this high availability of glucose to make these cells independent of oxygen by imposing the implementation of an anaerobic glycolytic metabolism.

The functional and metabolic shift observed in vitro in proximal tubule cells treated by GC7 leads to a decrease in glucose reabsorption by the kidney in vivo. Indeed, the increase in glucosuria highlights a deficit in glucose reabsorption and the increase in lactaturia is linked to the anaerobic glycolysis setting up along the nephron. This is reinforced by the fact that the lactate plasma level is not modified by GC7 treatment. Surprisingly the decrease in glucose uptake found in vitro was not linked to sglt expression alteration, while sglt glucose transport is required for PCT cells survival. SGLT co-transporters are electrogenic due to their coupling with Na^+^ and their activities depend both on the plasma membrane potential and the inward Na^+^ gradient, thus we suspect that GC7 treatment could modify the membrane potential and/or the inward directed sodium gradient resulting in an apparent independence between their expressions and transport capacities. We cannot exclude a more general effect of GC7 on whole-body metabolism that could be compensated by other physiological mechanisms. Hepatic clearance is the main mechanism regulating hyperlactatemia, in addition to other tissues as skeletal muscles, heart and kidney proximal tubule which clear lactate by converting it to pyruvate^28^. In vitro we demonstrate that proximal cells are unable to use lactate as substrate, suggesting that kidney cells are not involved in lactate clearance. We know that GC7 can perform this metabolic switch in other organs and cells such as the brain and neurons^15^. Therefore, considering our findings in kidney cells, it is tempting to assume that an epithelium with equivalent glucose metabolism may also be targeted and thereby protected by GC7.

In vitro we demonstrated that inhibition of eIF5A hypusination leads to a shift from mitochondrial oxidative phosphorylation to anaerobic glycolysis. Nevertheless, the pathway governing GC7 effect still needs to be clarified. Indeed, inhibition of mitochondrial activity could promote metabolic shift and favour anaerobic glycolysis leading to inhibition of glucose efflux to preserve cell integrity. The low level of PDH phosphorylation found in GC7-treated cells, demonstrating the inability of mitochondria to perform oxidative phosphorylation even in the presence of pyruvate, could suggest that the mitochondria “silencing” occurred before the onset of glycolysis. Another way would be that the increase in glucose availability, due to GLUT1 misexpression, enhances glucose metabolism concomitantly to inhibition of mitochondria activity, which ultimately leads to an anaerobic metabolism. In the context of an increased glycolysis a parallel point has also to be studied in the future: it is the effect of GC7 on the pentose pathway involving glucose 6 phosphate that is produced early in glycolysis and that provides carbon skeleton for the synthesis of nucleic acids. So many questions that deserve to be studied in order to fill in the gaps of the molecular mechanisms linking eIF5A hypusination inhibition to metabolic shift. Puleston et al.^16^ demonstrated that GC7 treatment of macrophages leads to an inhibition of several mitochondrial proteins explaining a shift to anaerobic glycolysis. These authors explained the decrease in a subset of mitochondrial proteins by a specific defect of their translation due to a specific motif hypersensitive to eIF5A hypusination^16^. These interesting results obtained in vitro involves that all cells displaying mitochondrial metabolism must be sensitive to GC7 treatment, which may not be the case especially in vivo were GC7 treatment did not imply a general shift toward anaerobic glycolysis in our model and others^9,13–15,29^.

It is known that *dhps* deficiency (the gene encoding DHS) or chronic treatment with DHS inhibitors, as GC7, ameliorate glucose tolerance and glycaemia in various mouse models of diabetes (HFD^30,31^, STZ^9,32^, humanized mouse model of T1D^33^, db/db^29^; NOD^34^). All these exciting studies relied hypusination inhibition effect to beta cell mass protection and decreased pancreas inflammation. Unfortunately, none of these works studied peripheral effects of GC7 and especially its impact on intestine and renal functions, as we demonstrated in this work. Thus, it will be very interesting to further analysed GC7 effect on glucose reabsorption functions in mouse models of diabetes in order to determine if eIF5A hypusination inhibition in this context could participate to glucose tolerance improvement. One of the more striking result of this study is the drastic inhibition of GLUT1 expression consecutive to GC7 treatment, an effect that is fully reversible in a couple of days and devoid of side effects in our time frame. Indeed, the physiological consequence of this misexpression leads to an inhibition of glucose reabsorption and an enhancement of glucosuria. This could be of importance in clinic since it has been shown that an antisense GLUT1 transgene could protect mesangial cells from glucose induction of GLUT1 and fibronectin expression that could be beneficial in the setting of diabetes^35^. Figure 8 summarizes the molecular effects we evidenced and that are driven by GC7 treatment regarding glucose handling. The remaining question is the link between eIF5A hypusination and GLUT1 expression. Indeed, GLUT1 amino acid sequence does not include proline-rich regions and its encoding mRNA sequence does not contain consensus sequences described up to now as having a link with eIF5A^3,36^. Thus, future investigations are warranted to establish additional target motifs and recognition sites of hypusinated eIF5A.

An important point is the complete reversion of the GC7 effect that demonstrates a non-harmful effect on the mitochondrial network but rather a transitory “silencing” effect. In conclusion, such a reversible pharmacological reprogramming of glucose handling and oxygen dependence by GC7 represents a pharmacological opportunity in ischaemic as well as hyperglycaemia-associated pathologies from the renal origin.

## Supplementary information

supplemental figure 1

supplemental figure 2

supplemental figure 3

supplemental figure 4

supplemental figure 5
