# Supplementary figures and images for: Inhibition of eIF5A hypusination reprogrammes metabolism and glucose handling in mouse kidney
Source: Cell Death Dis. 2021 Mar 17;12(4):283. doi: 10.1038/s41419-021-03577-z (PMC7969969; doi:10.1038/s41419-021-03577-z)

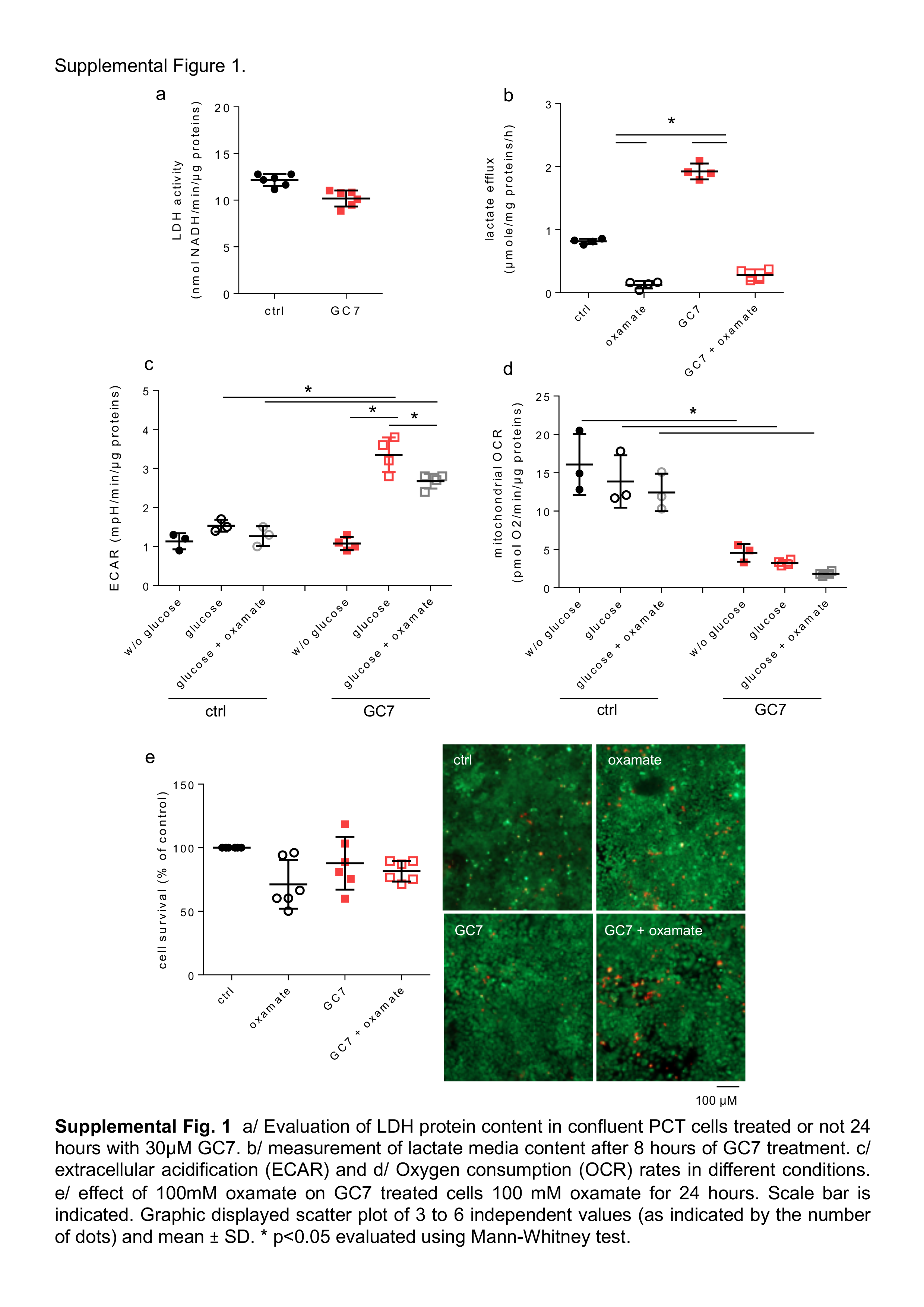

Supplement: Supplementary file 1 — supplemental figure 1 [file 41419_2021_3577_MOESM1_ESM.png]

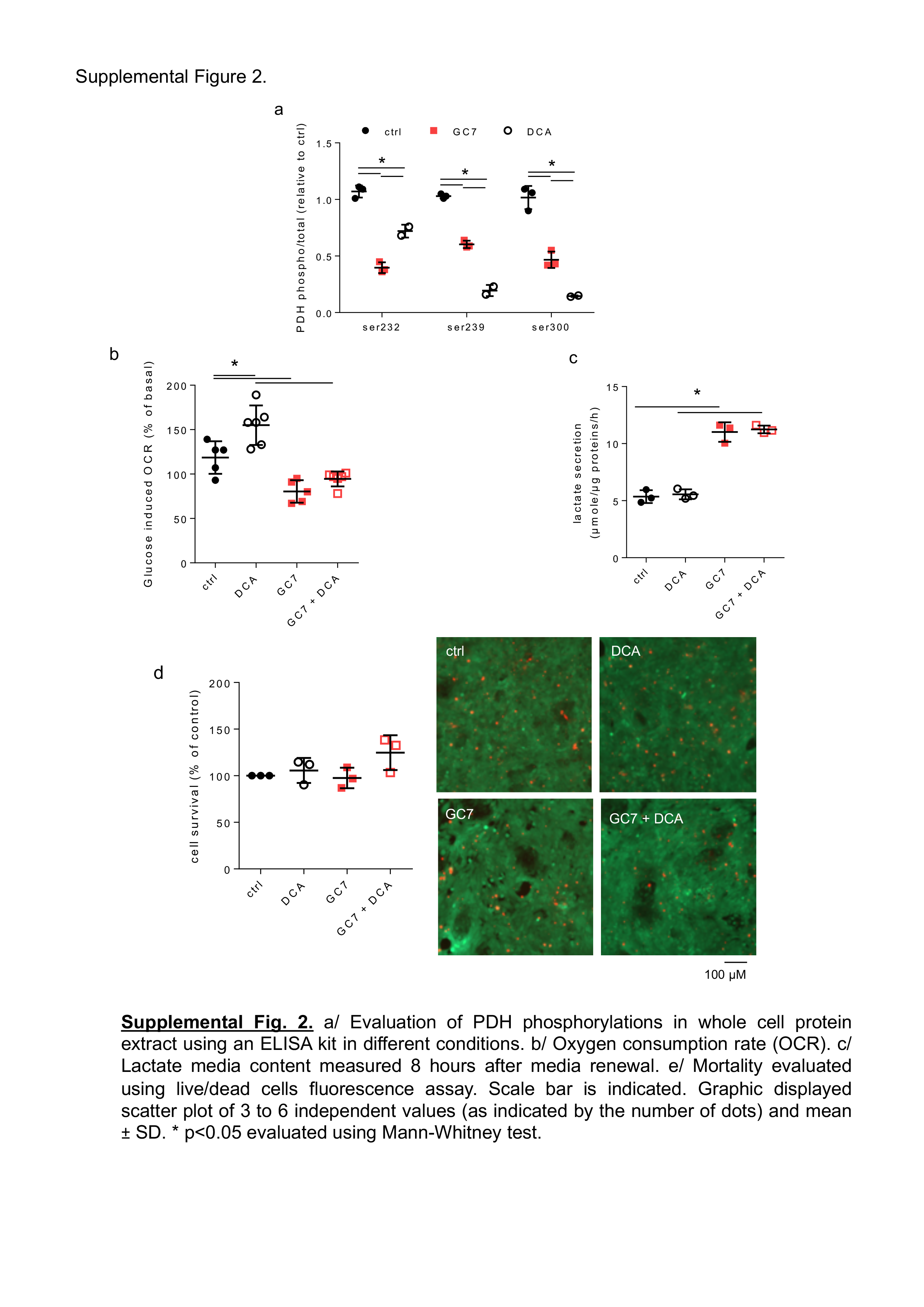

Supplement: Supplementary file 2 — supplemental figure 2 [file 41419_2021_3577_MOESM2_ESM.png]

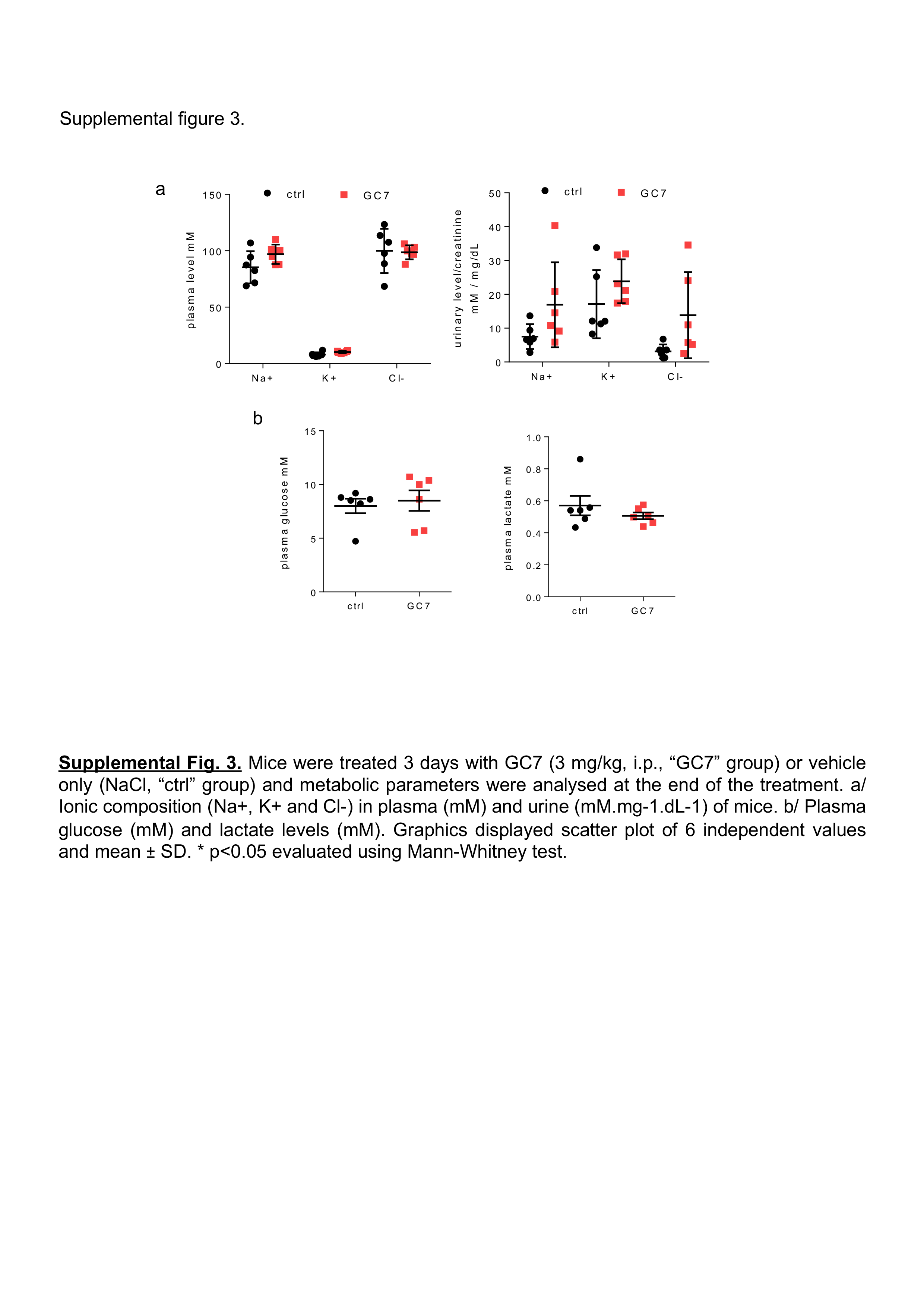

Supplement: Supplementary file 3 — supplemental figure 3 [file 41419_2021_3577_MOESM3_ESM.png]

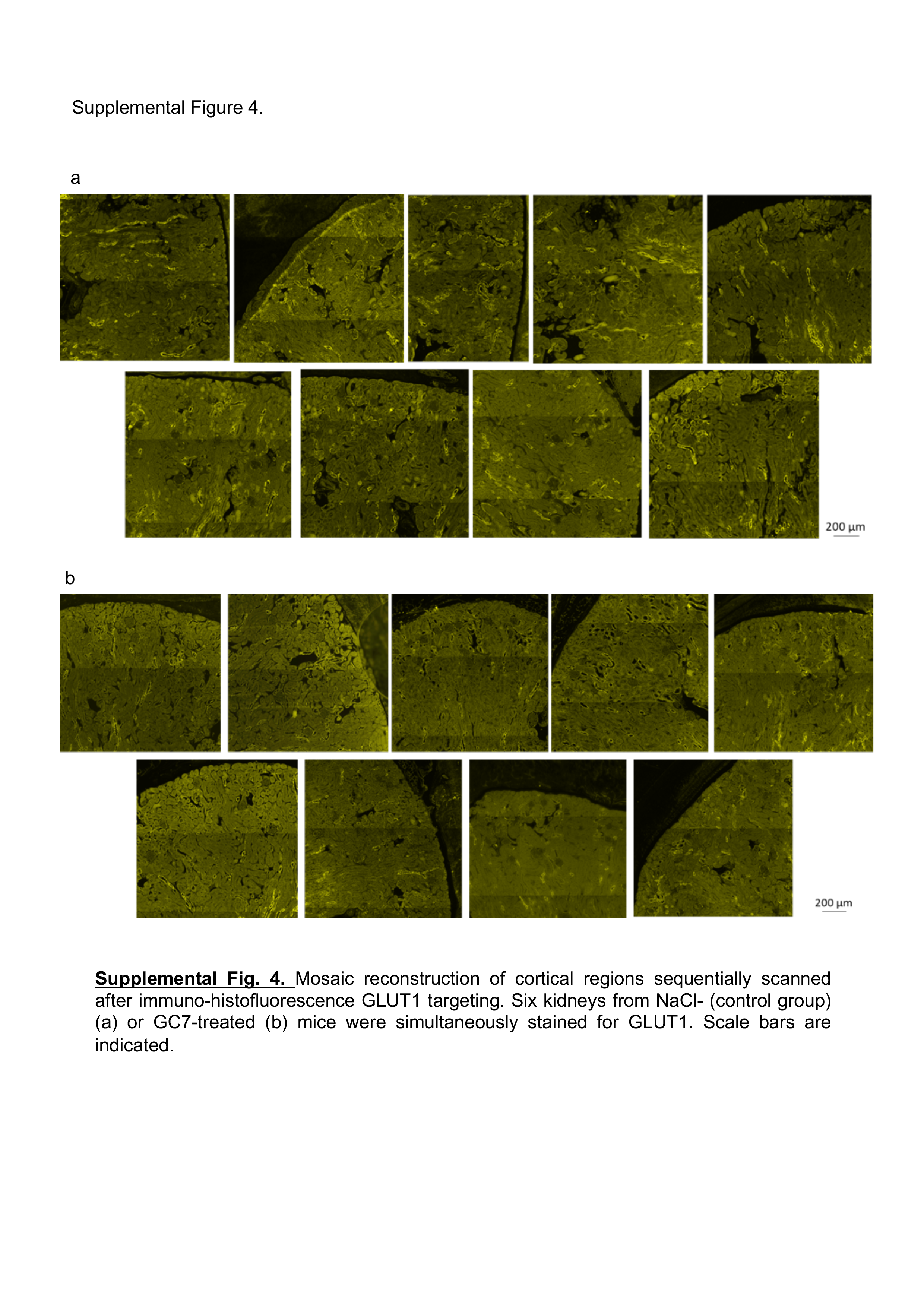

Supplement: Supplementary file 4 — supplemental figure 4 [file 41419_2021_3577_MOESM4_ESM.png]

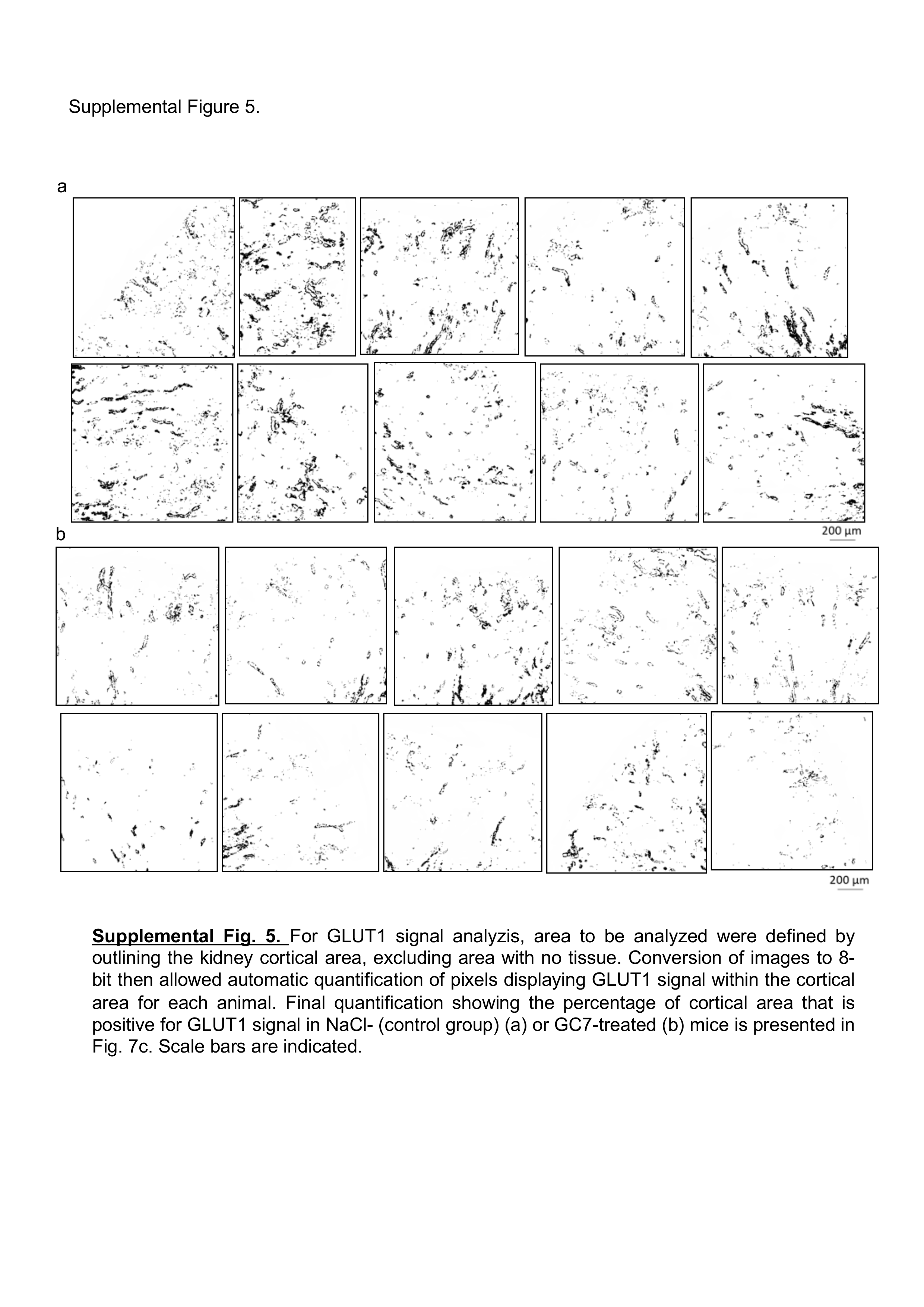

Supplement: Supplementary file 5 — supplemental figure 5 [file 41419_2021_3577_MOESM5_ESM.png]
